# Facial nerve reconstruction for flaccid facial paralysis: a systematic review and meta-analysis

**DOI:** 10.3389/fsurg.2024.1440953

**Published:** 2024-07-22

**Authors:** Friedemann Zumbusch, Peter Schlattmann, Orlando Guntinas-Lichius

**Affiliations:** ^1^ Department of Otorhinolaryngology, Jena University Hospital, Jena, Germany; ^2^ Department of Medical Statistics, Computer Sciences and Data Sciences, Jena University Hospital, Jena, Germany; ^3^ Facial-Nerve-Center, Jena University Hospital, Jena, Germany; ^4^ Center for Rare Diseases, Jena University Hospital, Jena, Germany

**Keywords:** facial nerve, hypoglossal nerve, masseteric nerve, facial reanimation, nerve suture

## Abstract

**Objectives:**

To determine the functional outcome after facial nerve reconstruction surgery in patients with flaccid facial paralysis.

**Methods:**

A systematic review and meta-analysis was performed on studies reporting outcomes after direct facial nerve suture (DFS), facial nerve interpositional graft suture (FIGS), hypoglossal–facial nerve suture (HFS), masseteric–facial nerve suture (MFS), and cross-face nerve suture (CFS). These studies were identified from PubMed/MEDLINE, Embase, and Web of Science databases. Two independent reviewers performed two-stage screening and data extraction. A favorable result was defined as a final House–Brackmann grade I–III and is presented as a ratio of all patients in percentage. Pooled proportions were calculated using random-effects models.

**Results:**

From 4,932 screened records, 54 studies with 1,358 patients were included. A favorable result was achieved after DFS in 42.67% of the patients [confidence interval (CI): 26.05%–61.12%], after FIGS in 66.43% (CI: 55.99%–75.47%), after HFS in 63.89% (95% CI: 54.83%–72.05%), after MFS in 63.11% (CI: 38.53%–82.37%), and after CFS in 46.67% (CI: 24.09%–70.70%). There was no statistically significant difference between the techniques (Q = 6.56, degrees of freedom = 4, *p* = 0.1611).

**Conclusions:**

The established facial nerve reconstruction techniques including the single nerve cross-transfer techniques produce satisfactory results in most of the patients with permanent flaccid facial paralysis. An international consensus on standardized outcome measures would improve the comparability of facial reanimation techniques.

## Introduction

Peripheral facial palsy is the most common pathology of cranial nerves with an incidence of 20–30 patients per 100,000 people per year (1). If the facial nerve is severed, for instance by nerve trauma, tumor infiltration, or tumor resection, the mimic muscles are denervated and spontaneous recovery is impossible. The result is a flaccid facial paralysis. This results in serious consequences for the patients: Insufficient corneal lubrication can lead to corneal ulceration and ultimately to a loss of vision (2). Furthermore, oral incompetence and facial asymmetry derive from facial paralysis (3). In addition, the psychosocial impairments are burdensome. The prevalence of anxiety and depression is significantly increased compared to a healthy population (4). Therefore, it is standard of care to perform a facial nerve reconstruction, if feasible, to reanimate the facial muscles (5).

**Figure 1:**
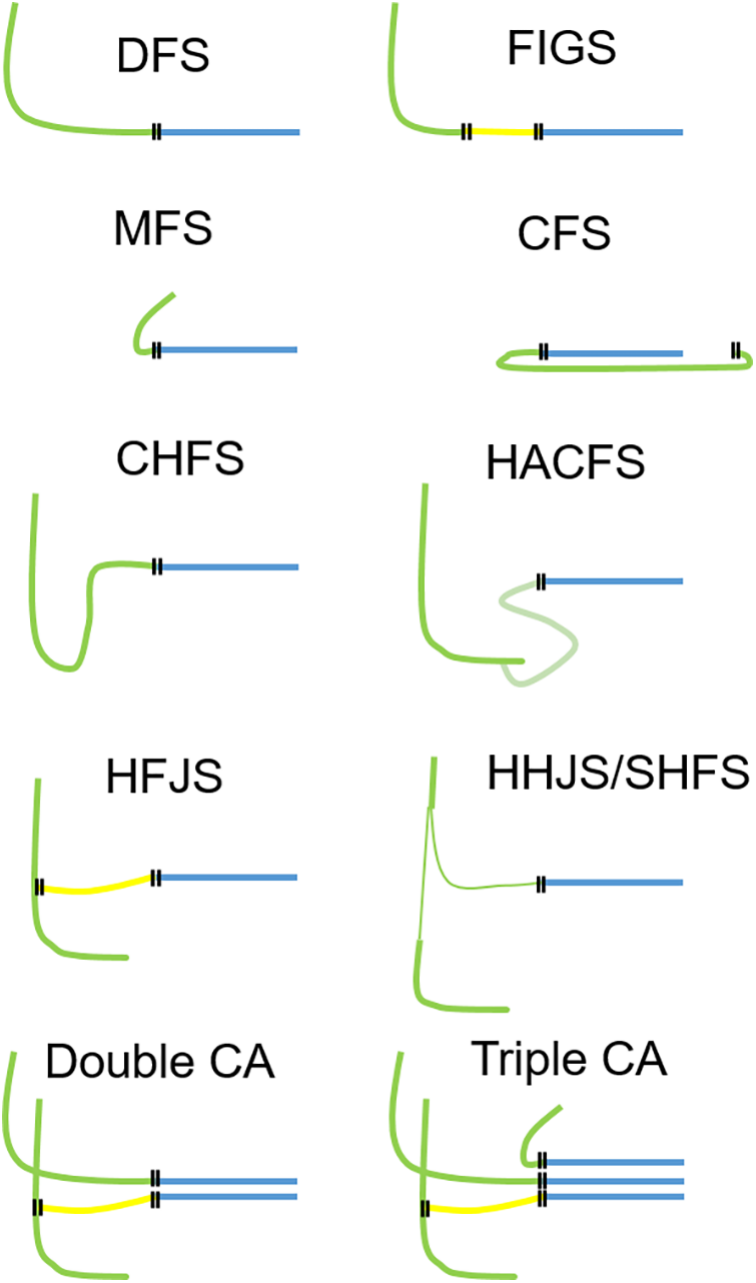
Overview of the most important facial nerve reconstruction techniques. Each technique can be used as a single measure, but also in combination (combined approach). Two examples for a combined approach are shown in the bottom row using two nerves for reanimation (left side) or even three nerves for reanimation (right side). Green, motor nerve used for facial reanimation; yellow, nerve graft; blue, peripheral facial nerve; each double line illustrates a suture site. DFS, direct facial nerve suture; FIGS, facial nerve interpositional graft suture; MFS, masseteric–facial nerve suture; CFS, cross-face nerve suture; CHFS, classical hypoglossal–facial nerve suture, HACFS, hypoglossal ansa cervicalis–facial nerve suture; HFJS, hypoglossal–facial jump nerve suture, HHFS, hemihypoglossal–facial nerve suture (HHFS); SHFS, split hypoglossal–facial nerve suture; CA, combined approach.

Surgical facial reanimation has been performed for over a century now. The first direct facial nerve repair was performed in 1884 by Sir Charles Alfred Ballance, a British aural surgeon, who published his experiences using this method in 1903 (6, 7). In 1895, Ballance performed the first facial-crossover nerve suture, using the accessorial nerve to reanimate the face. Neither of the surgeries succeeded, on the contrary the first patient died of sepsis (6, 7). The first successful hypoglossal–facial nerve suture (HFS), i.e., a cross-nerve suture technique, was performed by Werner Körte in 1901 (8). Since the first efforts to develop a sufficient technique to treat facial paralysis, plenty of articles have been published, showing different methods to achieve satisfactory results in facial reanimation. The most established techniques are direct facial nerve repair without a nerve graft, facial nerve interpositional graft, cross-face reanimation using the contralateral facial nerve and nerve grafts, a hypoglossal–facial nerve suture in different variations, and more recently masseteric–facial nerve suture (MFS) (Figure 1) (5, 9).

Since most of the available studies are relatively small in sample size, use only a single technique, and do not compare their methods to others, it is not clear which method for facial reanimation leads to the best results. In addition, there is a wide range of different scoring systems to report results after facial reanimation, which makes a comparison even more difficult (10).

Thus, the aim of the present study was to perform a meta-analysis to compare the results of the most used facial nerve reconstruction techniques in term of functional outcome.

## Material and methods

This study followed the Preferred Reporting Items for Systematic Reviews and Meta-Analyses (PRISMA) guidelines (11). Ethical approval and patient informed consent were not required for a meta-analysis.

### Data sources and literature search

Electronic databases (PubMed/MEDLINE, Embase, and Web of Science) were screened. The following Medical Subject Heading (MeSH) terms were used: (“facial palsy” OR “facial paralysis” OR “facial reanimation” OR “facial paresis”) AND (“hypoglossal nerve” OR “masseter nerve” OR “facial nerve” OR “nerve graft” OR “cross face” OR “accessory nerve”)”. The literature search revealed 4,932 results until the end of 2022.

### Selection of studies

Two independent reviewers (FZ and OG-L) reviewed the abstracts and full texts. If they came to a different conclusion, a joint decision was made in a discussion. All studies were assessed against the general exclusion criteria: review articles, duplicate patients, absence of essential data, multiple use of the same patient dataset, and animal studies. Further exclusion criteria were as follows: non-English or non-German language; full text not available; insufficient reported data or non-extractable data; case series including less than five patients; subgroup analyses of patients from larger studies; article types including reviews, case reports, conference abstracts, letters to the editor, or book chapters. No restrictions on the publication date were applied, but peer-reviewed journal publication was a requirement for article inclusion.

### Eligibility criteria

**Figure 2:**
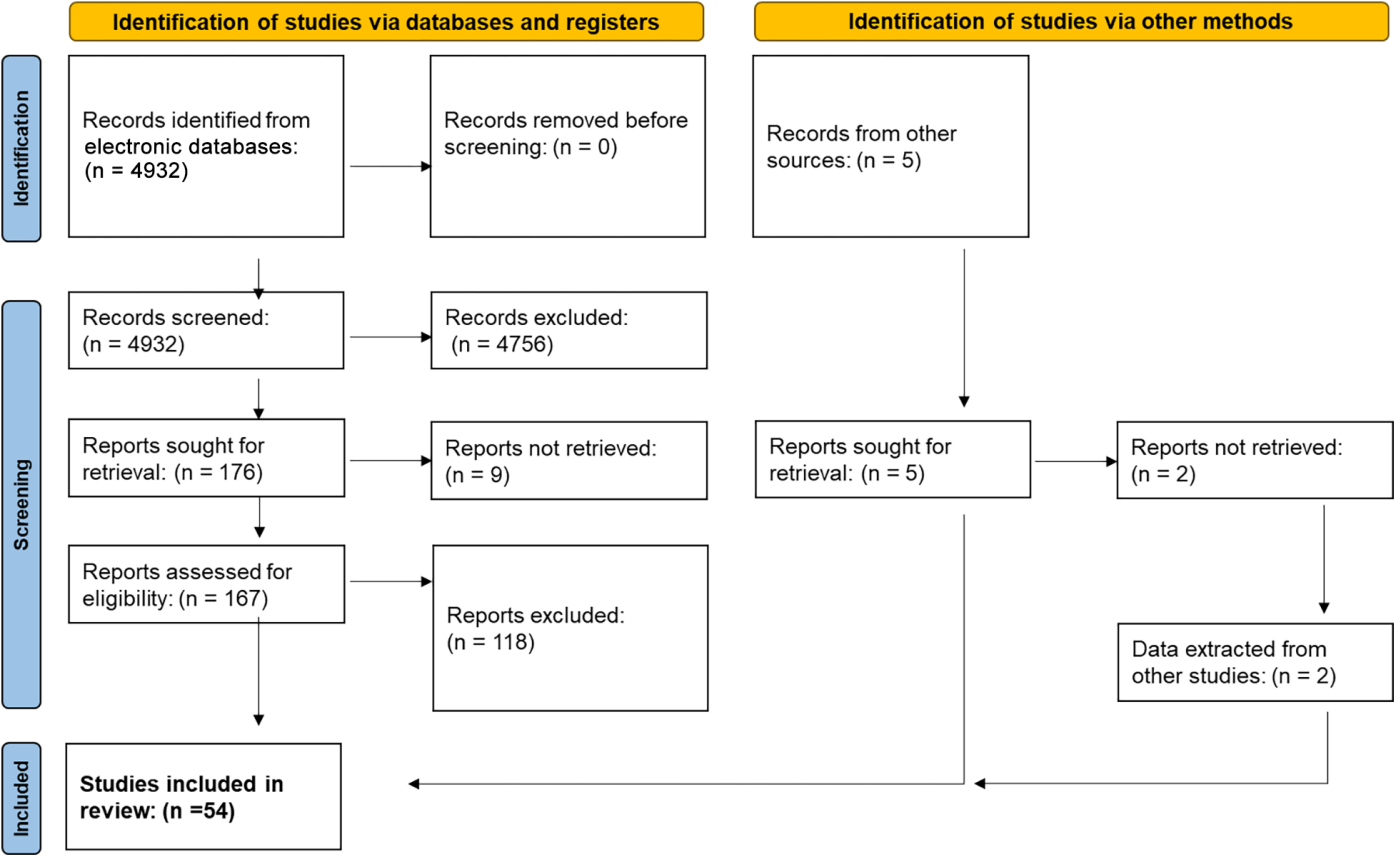
PRISMA flow diagram shows the selection of the included studies. PRISMA, Preferred Reporting Items for Systematic Reviews and Meta-Analysis.

The PICOS scheme was utilized to establish the eligibility criteria for this study, as follows: Patients (P), either children or adults with acquired unilateral complete facial paralysis; Intervention (I), reconstruction of the peripheral facial nerve; Comparison (C), comparison between the different reconstruction techniques; Outcomes (O), functional outcome of the reconstruction; Study design (S), retrospective and prospective cohort studies, case–control studies, case series, and randomized clinical trials (RCTs). Studies were included when they used a nerve-to-nerve neurorrhaphy for facial reanimation without muscle flap or other nerve transposition, contained at least five patients receiving the same reanimation technique, and used the House–Brackmann (HB) grading system to report the outcome (12). Four studies that used a modification of the HB score were also included. The results had to be reported according to patient data. Studies that used multiple reanimation methods without differentiating in their reported results were excluded. In total, 54 articles were finally included in the analysis (Figure 2).

### Data extraction

The following data were extracted from the included publications: number of patients, publication type, type of facial reconstruction surgery, HB score. The studies were pooled and sorted by the used reanimation technique into five different groups, as follows: hypoglossal–facial nerve suture, masseteric–facial nerve suture, cross-face reanimation, direct facial nerve repair with interpositional graft, and direct facial nerve repair without graft. The groups were then compared to each other to find out which technique had the best results. Results achieving an HB grade of I to III were defined as satisfactory results. The results are presented as the ratio of good results (HB grade I–III) to the total number of treated patients.

Since there are multiple different techniques using the hypoglossal nerve for reanimation, four additional subgroups were created to compare against each other. The compared techniques were the classical hypoglossal–facial nerve suture (CHFS) (end-to-end), hypoglossal–facial jump graft nerve suture (side-to-end using a jump interposition graft), hemihypoglossal–facial nerve suture (HHFS) (side-to-end without a graft), and split hypoglossal–facial nerve suture (splitting the hypoglossal nerve and using one-half to connect in an end-to-end manner to the facial nerve).

### Statistics

Statistical analysis was performed in R (version 4.0.4; www.r-project.org) (13). The meta package (version 4.16-2) was used to produce pooled estimates and forest plots (14). The proportion of favorable results in the treated patients is presented in the forest plots together with a 95% Clopper–Pearson confidence interval (CI) was used in this study. Assessment of the statistical heterogeneity was performed using Cochran's Q-test. The degree of heterogeneity was also quantified using I^2^ and using a random-effects model according to DerSimonian and Laird. Pooled estimates are derived from this model. To investigate potential differences between the applied techniques, subgroup analyses were performed. We employed either a fixed-effect or random-effects model depending on the calculated heterogeneity. We reported Q statistics, degrees of freedom (df), and associated *p*-values for these comparisons.

## Results

### Characteristics of the studies

The analysis included 54 publications with a total of 1,358 patients (Table 1). In total 118 studies were excluded. A reconstruction of the facial nerve using an interposition graft was performed in 481 patients, a reconstruction of the facial nerve without an interposition graft, in 47 patients. For facial reanimation with a cross-nerve technique, the hypoglossal nerve was used in 778 patients, the masseteric nerve in 37 patients, and a cross-face graft in 15 patients.

**Table 1:** Included studies in alphabetical order by first author^a^.

Study	Year	Study type	Number of patients	Surgery	Success rate: Proportion of HB I–III as final result
Arai et al. (15)	1995	Retrospective	8	HFS	1
Arriaga and Brackmann (16)	1992	Retrospective	8	FIGS	0.12
Arriaga and Brackmann (16)	1992	Retrospective	13	DFS	0.38
Beutner et al. (17)	2013	Retrospective	5	HFS	1
Blomstedt et al. (18)	1994	Retrospective	7	FIGS	0.57
Brudny et al. (19)	1988	Prospective	30	HFS	0.90
Catli et al. (20)	2010	Retrospective	35	HFS	0.71
Toffola et al. (21)	2014	Prospective	30	HFS	0.77
Darrouzet et al. (22)	1999	Retrospective	28	HFS	0.32
Donzelli et al. (23)	2005	Prospective	16	HFS	0.44
Dziedzic et al. (24)	2018	Prospective	57	HFS	0.84
Eby et al. (25)	1992	Retrospective	5	FIGS	1
Fagan and Loh (26)	1989	Retrospective	6	HFS	0.17
Fagan and Loh (26)	1989	Retrospective	5	FIGS	0.4
Falcioni et al. (27)	2003	Retrospective	56	FIGS	0.46
Fisch et al. (28)	1987	Retrospective	8	FIGS	1
Flores (29)	2007	Retrospective	8	HFS	0.62
Godefroy et al. (30)	2007	Retrospective	7	HFS	0.86
Green et al. (31)	1994	Retrospective	9	FIGS	0.22
Günther et al. (32)	2010	Retrospective	21	FIGS	0.86
Hammerschlag (33)	1999	Prospective	17	HFS	0.88
Han et al. (34)	2017	Prospective	14	HFS	0.64
Husseini et al. (35)	2013	Retrospective	40	HFS	0.65
Kunert et al. (36)	2011	Retrospective	7	HFS	1
Kunihiro et al. (37)	1996	Retrospective	29	HFS	0.24
Kunihiro et al. (38)	2003	Retrospective	42	HFS	0.4
Laskawi (39)	1997	Prospective	10	HFS	0.6
Le Clerc et al. (40)	2013	Retrospective	36	HFS	0.53
Leonetti et al. (41)	2007	Retrospective	40	FIGS	0.82
Linnet and Madsen (42)	1995	Retrospective	32	HFS	0.25
Luetje et al. (43)	1991	Retrospective	19	DFS	0.37
Magliulo et al. (44)	2001	Retrospective	14	HFS	0.36
Magliulo et al. (44)	2001	Retrospective	6	FIGS	0.67
Manni et al. (45)	2001	Retrospective	29	HFS	0.66
Martins et al. (46)	2008	Retrospective	36	HFS	0.72
Matejcik and Penzesova (47)	2008	Retrospective	10	HFS	1
Matsuda et al. (48)	2015	Retrospective	11	FIGS	0.64
Matsunaga et al. (49)	1995	Retrospective	10	HFS	0.5
Mohamed et al. (50)	2016	Retrospective	11	HFS	0.82
Mohamed et al. (50)	2016	Retrospective	11	FIGS	0.73
Okochi et al. (51)	2018	Retrospective	15	CFS	0.47
Ozmen et al. (52)	2011	Retrospective	155	FIGS	0.68
Rebol et al. (53)	2006	Retrospective	5	HFS	0.4
Rochkind et al. (54)	2008	Retrospective	13	HFS	0.69
Saeed and Ramsden (55)	1996	Retrospective	8	HFS	0
Saeed and Ramsden (55)	1996	Retrospective	9	DFS	0.78
Saeed and Ramsden (55)	1996	Retrospective	12	FIGS	0.83
Sakthivel et al. (56)	2020	Prospective	6	MFS	0.5
Samii et al. (57)	1985	Retrospective	27	FIGS	0.74
Samii et al. (58)	2015	Retrospective	26	HFS	0.73
Samii and Matthies (59)	1994	Retrospective	74	HFS	0.74
Samii and Matthies (59)	1994	Retrospective	61	FIGS	0.69
Sforza et al. (60)	2014	Prospective	14	MFS	0.86
Shipchandler et al. (61)	2011	Prospective	13	HFS	0.69
Slattery et al. (62)	2014	Retrospective	19	HFS	0.37
Sood et al. (63)	2000	Retrospective	29	HFS	0.66
Stephanian et al. (64)	1992	Retrospective	22	FIGS	0.45
Venail et al. (65)	2009	Retrospective	12	HFS	0.5
Wang et al. (66)	2013	Retrospective	12	HFS	0.67
Wang et al. (66)	2013	Retrospective	13	FIGS	0.77
Yammine et al. (67)	1999	Retrospective	4	FIGS	0.5
Yammine et al. (67)	1999	Retrospective	6	DFS	0.17
Zotov et al. (68)	2016	Retrospective	17	MFS	0.47

HFS, hypoglossal–facial nerve suture; MFS, masseteric–facial nerve suture; FIGS, facial nerve interpositional graft suture; DFS, direct facial nerve suture; CFS, cross-face nerve suture.

^a^ If several facial nerve reconstruction techniques were analyzed in the same study, each technique is presented separately.

### Functional outcome of the different reconstruction techniques

**Figure 3:**
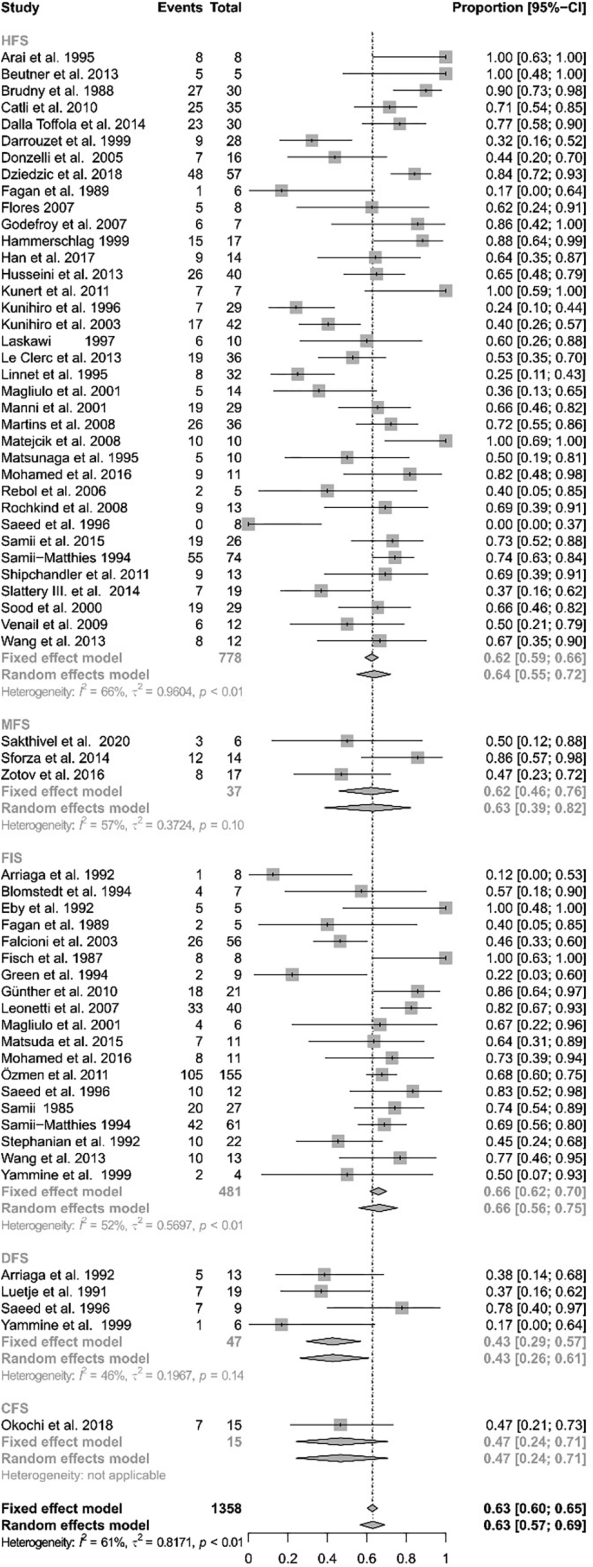
Forest plot illustrating the rates of successful facial reanimation for hypoglossal–facial nerve suture (HFS), masseteric–facial nerve suture (MFS), facial nerve interpositional graft suture (FIGS), direct facial nerve suture (DFS), and cross-face nerve suture (CFS). The proportion of success can reach from 0 (no patient with HB I–III as final result) to 1 (all patients reached a HB I-III as a final result).

Figure 3 gives an overview on the results of all the analyzed reconstruction techniques. The analysis showed significant heterogeneity (I^2^ = 61.1%, Q = 247.37, df = 62, *p* < 0.0001). Thus, a random-effects model was used. A direct facial nerve reconstruction achieved good results in 42.67% of the patients (CI: 26.05%–61.12%). A facial nerve reconstruction with an interpositional graft had good results in 66.43% (CI: 55.99%–75.47%). The facial reanimation using the hypoglossal nerve achieved good results in 63.89% (95% CI: 54.83%–72.05%). The use of the masseteric nerve achieved good results in 63.11% (CI: 38.53%–82.37%). Finally, a facial reanimation using a cross-face technique achieved good results in 46.67% (CI: 24.09%–70.70%). While the direct facial nerve repair exhibited the lowest proportion of good results, the random-effects model revealed no statistically significant differences between the groups (Q = 6.56, df = 4, *p* = 0.1611).

**Figure 4:**
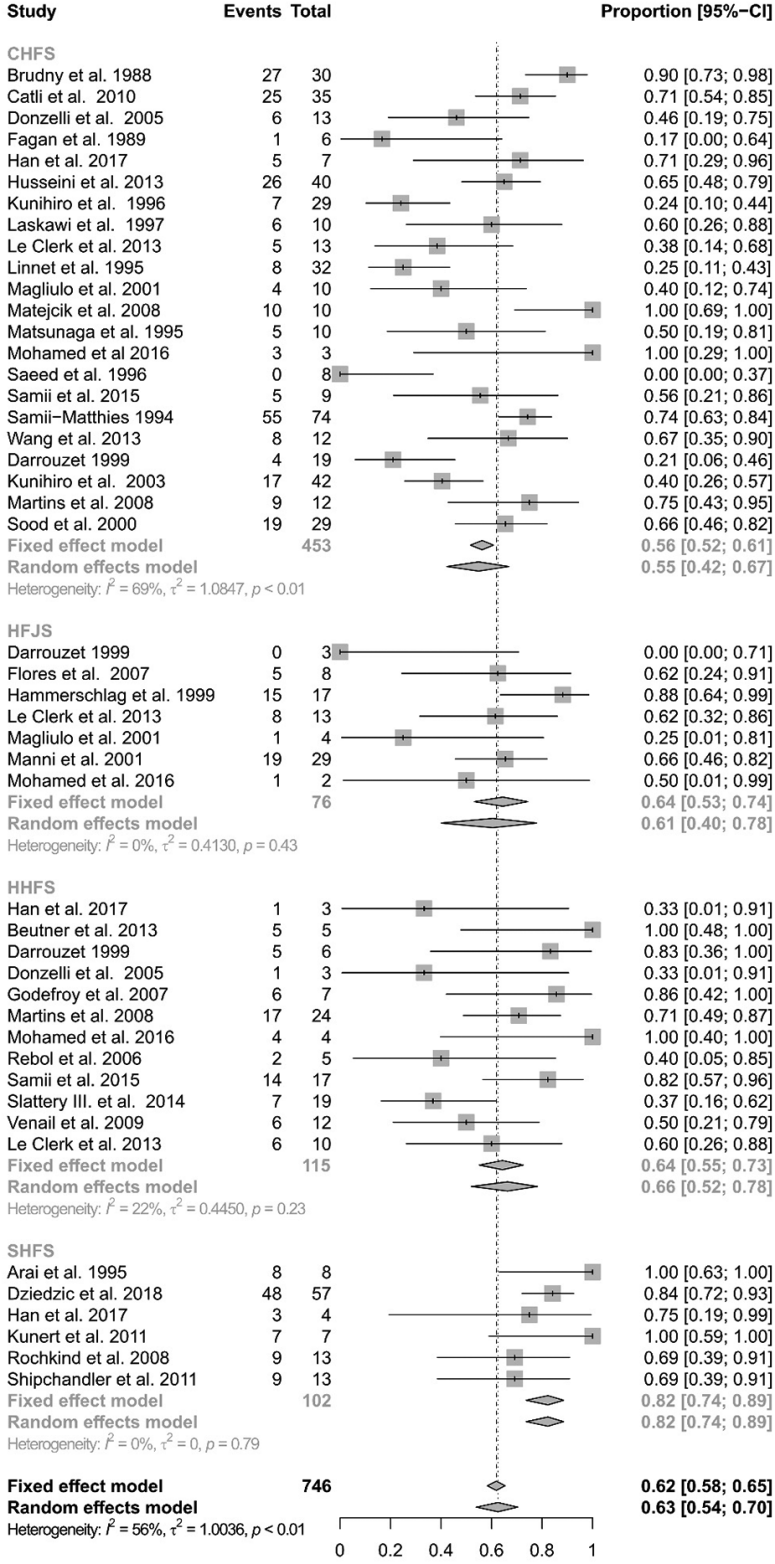
Forest plot illustrating the rates of successful facial reanimation for classical hypoglossal–facial nerve suture (CHFS), hypoglossal–facial jump nerve suture (HFJS), hemihypoglossal–facial nerve suture (HHFS), and split hypoglossal–facial nerve suture (SHFS). The proportion of success can reach from 0 (no patient with HB I-III as a final result) to 1 (all patients reached a HB I-III as a final result).

Figure 4 shows the subanalysis on the different techniques using the hypoglossal nerve as cross-motor nerve for the peripheral facial nerve reanimation. Out of the 778 reconstructions using the hypoglossal nerve, there were 453 classical, 76 jump graft, 115 hemihypoglossal, and 102 split hypoglossal-to-facial nerve reconstructions. The specific technique was not reported for 32 patients. Here again, the analysis showed significant heterogeneity (I^2^ = 55.9%, Q = 184.56, df = 46, *p* < 0.0001) and consequently a random-effects model was used. The classical hypoglossal–facial nerve suture achieved satisfactory results in 54.90% of the patients (CI: 42.33%–66.87%). The jump-graft technique achieved satisfactory results in 60.53% (CI 40.07%–77.87%). The hemihypoglossal technique achieved satisfactory results in 66.35% (CI: 52.04%–78.18%). Finally, the reanimation using a split hypoglossal nerve achieved satisfactory results in 82.35% (CI: 73.72%–88.59%). There was a statistically significant difference between methods, when using a random-effects model (Q = 14.48, df = 3, *p* = 0.0023). Hence, the split hypoglossal nerve technique presented the best results among the hypoglossal nerve cross-motor techniques.

## Discussion

This meta-analysis comparing different surgical techniques for facial nerve reconstruction for patients with permanent facial paralysis did not show a significant higher succession rate for one of the compared techniques. Numerous studies have explored surgical methods for facial reanimation, yielding results that vary and, in some cases, conflict with one another (66, 69–74). Nevertheless, a meta-analysis conducted on a large scale that compares the various approaches and techniques has not been done yet. There is one recent meta-analysis dealing only with masseteric nerve transfer and time to first movements as outcome measure, showing that such a transfer shows overall good results (3). Urban et al. also compared hypoglossal and masseteric nerve transfer for facial reanimation in a meta-analysis (75). Here, the outcome measure was oral commissure symmetry, time to reinnervation, and Sunnybrook grading. Both techniques achieved good results, but the masseteric nerve transfer overall showed better results.

Most data were available for the reconstruction using the hypoglossal nerve. The use of the hypoglossal nerve is the oldest standard cross-nerve reconstruction technique in case of long-term denervation (1, 9, 76). The cross-nerve techniques with the hypoglossal nerve, the masseteric nerve, or with branches of the contralateral facial nerve are mainly used for patients with permanent flaccid facial paralysis or in case of immediate facial nerve reconstruction if the proximal facial nerve stump is not available. The reconstruction using the hypoglossal nerve, the masseteric nerve, and the facial nerve reconstruction using an interposition graft achieved similar results. The latter is only feasible when a proximal facial nerve stump is available. A typical example is a complex defect of the extratemporal facial plexus after resection of a parotid tumor with facial nerve infiltration (77).

The facial reanimation using a direct facial nerve reconstruction or a cross-face nerve graft tended to achieve the worst results compared to the other techniques. A direct facial nerve reconstruction is typically only feasible for a sharply cut nerve, for instance in case of an iatrogenic lesion or after facial nerve trauma if immediate repair is possible. It is noteworthy that the data for the cross-face graft were extracted from a single study, in which a special emphasis was put on the reanimation of the periorbital movement, thus limiting the validity for general facial reanimation. Then, cross-face grafts are often limited to reanimation of the lower face. Lastly, a possible explanation for the worse results might be the relatively extended length of the interposition grafts, which is discussed to be a negative predictor of the results of facial reanimation (31).

Comparing the different available techniques for facial reconstruction using the hypoglossal nerve we found that the best results were achieved using a split hypoglossal technique. Due to its wider diameter compared to the facial nerve, half of the hypoglossal nerve provides a sufficient number of axons to enable facial reanimation. In addition, employing the split hypoglossal technique allows axons sprouting in their natural direction. Furthermore, the positive outcomes may be attributed to the use of a single nerve suture without the need for an interposition graft as it is necessary for the hypoglossal–jump nerve alternative (61). When using the masseteric nerve, main trunk coaptation without interpositional grafts results in faster reinnervation than in reconstruction with interpositional graft (3). If faster reinnervation correlates with functionally, better reinnervation is not proven yet.

This study has a number of limitations. There is a wide range of factors that might have influenced the result of facial reanimation procedures. Some of them include the time to reanimation, the patients’ age, graft length, the surgeon's experience, reason for palsy, post-operative rehabilitation process, and comorbidities (21, 27, 31, 78, 79). Those factors could not be assessed in the current study. Another limitation is the way the results of facial nerve were reported. The HB grading system had to be used as an outcome measure for the post-surgical facial nerve function, because there are no sufficient number of studies with more modern grading systems that would permit a meta-analysis. The HB grading system is a very gross system and not very reliable (80). Small differences or advantages of the used techniques might not be detected by the HB grading. Thus, results like spontaneous smile might not properly be displayed by the HB grading. The HB system had to be selected because most studies used it. Only in newer studies, more reliable but still subjective systems like a Sunnybrook or eFACE grading are used (75, 76). A wider use of objective automated image analysis tools for evaluation of the surgical outcome would be better (81, 82). Then, the donor site morbidity could not be analyzed. Especially, the harvest of donor nerves needed as grafts for some of the facial nerve reconstruction techniques, could lead to additional morbidity. The morbidity was often not measured at all, let alone in a standardized way. For instance, the classical HFS was abandoned in favor of the jump or split technique in many centers due to the severe morbidity, as the patients suffered greatly from the tongue palsy in the long term. Furthermore, only a limited number of studies also address the quality of life of the patients using facial-specific patient-reported outcome measures. It is recommended to use patient-reported outcome measures like the Facial disability index (FDI) or the Facial Clinimetric Evaluation Scale (FaCE) to record the patient's view of the surgical result (70, 76, 83).

Since reconstruction using a combination of multiple techniques (dual or even triple innervations) or muscle transplantations were ruled out in the present study to define the effects of a single nerve for reanimation, newer methods using multiple nerves were systematically excluded, even though they might achieve better results (84–87).

## Conclusion

In conclusion, all commonly used facial nerve reconstruction techniques are a viable option for facial reanimation for patients with permanent flaccid facial paralysis. The outcome in these patients should be measured with standardized and reliable outcome parameters. Highly reliable grading systems and facial-specific quality-of-life assessment should be used on all these patients. The introduction of an objective automated image analysis tool for a comprehensive quantification of the outcome would be perfect.

## Data Availability

The raw data supporting the conclusions of this article will be made available by the authors, without undue reservation.
